# Hooping Cough

**Published:** 1845-10

**Authors:** 


*Hooping Cough.*—A correspondent at Ipswich says—Seeing some useful remarks by Mr. Waddington, in the Lancet for June 21st, on this distressing complaint among children, I beg to call his attention, as well as that of the profession generally, to the speedy relief afforded by the following simple remedy, viz: from fifteen to twenty drops of diluted sulphuric acid, P.L., mixed in a teaspoonful of moist sugar, taken three or four times a day. I sometimes prefer giving an ounce of this "elixir" in a pint of water, with two ounces of simple syrup; the dose, a tablespoonful three or four times a day. This popular remedy has been found so useful here, during the last two or three years, as to be considered almost a specific. Permit me also to take the opportunity of calling the attention of the profession to the great utility of emetics, particularly sulphate of zinc, in all cases of *asphyxia*, or suspended animation, as well as in convulsions.—*Boston Med. and Surg. Journal*, from *London Lancet.*

